# Perforated Intestine Searcher

**Published:** 1901-10

**Authors:** Lucian Lofton

**Affiliations:** Emporia, Va., Ex-President Seaboard Medical Association of Virginia and North Carolina


For Texas Medical Journal.

### Perforated Intestine Searcher.

BY LUCIAN LOFTON, A. B., M. D., EMPORIA, VA.,
Ex-President Seaboard Medical Association of Virginia and North Carolina.

I wish to present to the medical profession a simple and effective device by which perforations of the human intestines may be diagnosticated.

This instrument has been used by the author with great practical success.

It is nothing more than a smoothly moulded glass tube, twenty-four inches in length, one-half inch in circumference, expanded at one end not unlike an ordinary funnel, two inches across at the top two inches in depth and gradually narrowing to the size of the tube proper. At its distal extremity it is well rounded and conical in appearance, and is practically the same size as the body of the rod.

In suspected cases of perforation, the tube may be heated over a benzine burner, alcohol lamp or otherwise, as convenience may dictate, and bent, at any angle desirable. Being made surgically sterile by repeated immersion in any good mild antiseptic solution, with a subsequent bath in proof spirit, you insert in the mouth or funnel a small, loosely fitting piece of sterile wool or dry absorbent cotton, and carefully permitting the tube to descend into the abdominal cavity, when by continued application of your nose to the funnel, should perforation exist, you will almost immediately get the aroma of escaping intestinal fluids. This, of course, will prove beyond a peradventure that perforation exists. Should you not succeed at once, to obtain the scent of escaping fecal matter, gentle manipulation to various parts of the cavity will reward you with success as to whether you have a lacerated bowel with which to deal.

*Never* use the instrument in the capacity of a probe, for it is agreed by most all leading surgeons that probing is not only obsolete, but dangerous, and withal practically useless.

After an examination, owing to the cheapness of the tube it may be destroyed.

In experiments upon dogs, rabbits and pigs this instrument has acted with every degree of success. Likewise upon one human being.

There is no distress following the exploration, and with adequate precautionary measures no sepsis will result.

I believe this tube to be more accurate in making diagnosis than anything heretofore offered the profession.

The precautions which may be guarded against are worthy of mention, viz.: Do not let the distal end of the tube rest upon the walls of the cavity at any time, as this will sometimes clog the aperture and thus defeat your object. Nor it is advisable to remove the instrument until you have completed your exploration.

The contents of the stomach, *per se*, have not been tested so far upon the human subject, but upon animals it has worked most admirably.

You can procure the instrument as outlined from any dealer on short notice.

